# Temperature stress-induced production of salicylic acid from endophytic *Aspergillus flavus* with its *in silico* and *in vitro* anti-inflammatory potential and promising effect in combination with ibuprofen

**DOI:** 10.3389/fmicb.2026.1777668

**Published:** 2026-04-29

**Authors:** Ayesha Saleem, Sajjad Ahmad, Saeed Ullah Khattak, Huma Jalil, Mostafa A. Abdel-Maksoud, Ahmed Othman Alsabih, Wahidah H. Al-Qahtani, Ahmed M. Hussein

**Affiliations:** 1 Center of Biotechnology and Microbiology (COBAM), University of Peshawar, Peshawar, Pakistan; 2 Department of Health and Biological Sciences, Abasyn University, Peshawar, Pakistan; 3 Research Chair of Biomedical Applications of Nanomaterials, Biochemistry Department, College of Science, King Saud University, Riyadh, Saudi Arabia; 4 Department of Physiology, College of Medicin, King Saud University, Riyadh, Saudi Arabia; 5 Department of Food Sciences and Nutrition, College of Food and Agricultural Sciences, King Saud University, Riyadh, Saudi Arabia; 6 Division of Pharmacology and Toxicology, Department of Pharmaceutical Sciences, University of Vienna, Vienna, Austria

**Keywords:** cyclin-dependent kinase 2 (CDK2), FTIR, fungal natural anti-inflammatory compound, GC–MS, molecular docking, NMR, protein denaturation inhibition

## Abstract

The present study investigates temperature stress-induced production of a salicylic acid derivative from endophytic *Aspergillus flavus* isolated from *Rosmarinus officinalis* (rosemary), with a focus on the potential of controlled temperature variations to enhance fungal-derived salicylic acid biosynthesis. Following morphological and microscopic identification, *A. flavus* was cultured in modified Czapek broth at temperatures ranging from 25°C to 42°C with 3°C stepwise increases in a shaking incubator at 150 rpm. Salicylic acid was extracted using ethyl acetate and purified through column chromatography, preparative high-performance liquid chromatography (HPLC), and gas chromatography–mass spectrometry (GC-MS), yielding 7.27 mg of pure compound from 81.39 g of crude extract in a 1000 ml culture volume. Structural confirmation was performed using Fourier-transform infrared spectroscopy (FTIR) and proton nuclear magnetic resonance (^1^H NMR) spectroscopy. Molecular docking studies demonstrated the highest binding affinity with cyclin-dependent kinase 2 (CDK2) (−6.1), followed by cyclooxygenase-1 (COX-1) (−6.0), cyclooxygenase-2 (COX-2) (−5.5), and high-mobility group protein B1 (HMGB1) (−4.9), indicating CDK2 as a potential primary target. *In vitro* anti-inflammatory activity, assessed using the egg albumin denaturation assay, showed that ibuprofen exhibited greater inhibition than salicylic acid alone; however, their combination resulted in the highest inhibition rate (29.56% at 300 μg mL^-1^), with a dose-dependent increase observed. These findings suggest that fungal-derived salicylic acid enhances the anti-inflammatory potential of ibuprofen and may serve as a promising natural adjunct to conventional nonsteroidal anti-inflammatory drug therapy.

## Graphical abstract

**Figure:**
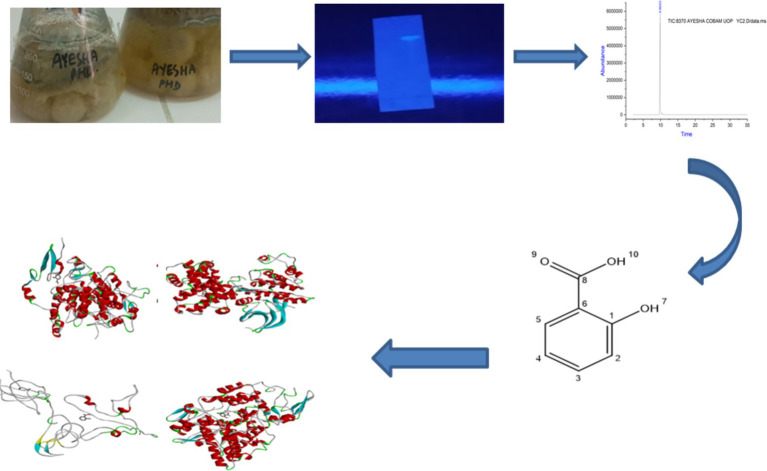

## Introduction

Inflammation is a natural and essential response of the innate and adaptive immune systems to infections, injuries, or harmful stimuli. However, when inflammation is not properly regulated, it can contribute to the development of chronic inflammatory conditions, including neurodegenerative diseases, autoimmune disorders, and even cancer. Over the past few decades, the global incidence of inflammatory diseases has risen significantly, posing major challenges due to prolonged treatment regimens and the increasing financial burden on healthcare systems (Dinarello, 2010; Ratjen and Bell, 2021; Peng et al., 2021). To manage inflammatory conditions, anti-inflammatory agents, either synthetic or naturally derived, are widely used. Synthetic anti-inflammatory drugs, including both steroidal and non-steroidal anti-inflammatory drugs (NSAIDs), are highly effective. However, their long-term use is associated with serious side effects, high costs, and limited accessibility. In contrast, natural anti-inflammatory compounds, extracted from plants and microbes, often exhibit comparable therapeutic potential with fewer side effects, making them a promising alternative (Yatoo et al., 2018).

Medicinal plants have been used for centuries to treat inflammatory disorders. Various herbs, such as *Zingiber officinale* (ginger), *Rosmarinus officinalis* (rosemary), *Borago officinalis*, *Salvia officinalis*, *Ribes nigrum*, and *Vaccinium myrtillus*, possess well-documented anti-inflammatory properties and have contributed to the development of plant-derived therapeutic agents. Several anti-inflammatory drugs derived from plant sources have even been patented, highlighting their pharmaceutical significance (Yatoo et al., 2018; Virshette et al., 2019; Akhtar, 2022). Salicylic acid is a naturally occurring organic compound with broad therapeutic applications, serving as a precursor for the synthesis of widely used drugs, including aspirin (2-Acetoxybenzoic acid), a landmark NSAID (Sambyal and Singh, 2021; Wu et al., 2018). Since the late 19th century, salicylic acid and its derivatives have remained among the most important drug classes, being extensively used for treating pain, fever, and inflammation. Despite their long history in medicine, researchers continue to explore new salicylic acid based compounds with improved efficacy, reduced side effects, and novel biological activities (Shchegol'kov et al., 2017; Ekinci et al., 2011). At the commercial level, salicylic acid and its derivatives are predominantly produced through chemical synthesis, where modifications with various functional groups enhance their therapeutic properties. However, in recent years, increasing attention has been given to microbial biosynthesis of salicylic acid, as certain bacterial and fungal species have been identified as natural producers (Shchegol'kov et al., 2017; Cipolla et al., 2002; Schadler and George, 2006). Several bacterial genera, including *Pseudomonas*, *Bacillus*, *Azospirillum*, *Salmonella*, *Yersinia*, *Achromobacter*, *Vibrio*, and *Mycobacterium*, have been reported to produce salicylic acid and its derivatives (Sambyal and Singh, 2021; Lockhart and Guarner, 2019). However, salicylate hydroxylase, an enzyme involved in salicylic acid metabolism, has been predominantly purified from fungal sources (Ambrose et al., 2015). Interestingly, plants also show a significant increase in salicylic acid biosynthesis in response to chemical, pathogen-induced, or environmental stress, indicating a strong link between stress conditions and salicylic acid production (Sambyal and Singh, 2021; Yalpani et al., 2001).

Building on these insights, our study explored the biosynthetic potential of *Aspergillus flavus*, an endophytic fungus isolated from *Rosmarinus officinalis* (rosemary). We investigated how temperature stress affects salicylic acid production, using controlled environmental conditions to enhance its biosynthesis. This study highlights how temperature variations influence fungal-derived salicylic acid production, along with its purification, structural characterization and anti-inflammatory activity assessed through both *in silico* and *in vitro* approaches.

## Methodology

### Isolation of endophytic fungi from rosemary (*Rosmarinus officinalis*)

Healthy tissues of *Rosmarinus officinalis*, including leaves, roots and twigs were collected from the botanical garden of University of Peshawar (UoP) in sterile zipper bags and transported to the Centre of Biotechnology and Microbiology, UoP for further processing. The samples were stored at −80 °C until use, for the isolation of endophytic fungi. The collected plant samples were initially sterilized to eliminate surface microorganisms. The sterilization process involved sequential washing with distilled water, followed by treatment with 75% ethanol for 1 min and 0.1% mercuric chloride solution for 1 min. After sterilization, the plant tissues were aseptically cut into 1 cm segments using a sterile blade and placed on Potato Dextrose Agar (PDA) medium supplemented with 50 μg mL^−1^ chloramphenicol to prevent bacterial contamination. The inoculated plates were sealed with parafilm and incubated at different temperature ranges from 18 to 28 ± 2 °C for 7 to 15 days to allow fungal growth. Once mature fungal colonies appeared, they were sub-cultured onto fresh PDA plates to obtain pure isolates for further identification (Sharma et al., 2016; Su et al., 2014).

### Identification of the endophytic fungal isolate

The endophytic fungal isolate obtained from *R. officinalis* was characterized through integrated morphological, microscopic, and molecular approaches. For phenotypic and microscopic examination, colony characteristics, hyphal organization, and spore morphology were assessed using the slide culture method. Preparations were stained with lactophenol cotton blue and observed under a light microscope at 1000 × magnification to record diagnostic traits, including septate hyphae and reproductive structures.

At the molecular level, genomic DNA was extracted from the pure culture using the Qiagen DNeasy Mini Kit, and its integrity was verified by 1% agarose gel electrophoresis. The Internal Transcribed Spacer (ITS) region of rDNA was amplified using universal fungal primers ITS1 (5′-TCCGTAGGTGAACCTGCGG-3′) and ITS4 (5′-TCCTCCGCTTATTGATATGC-3′). Sequencing was performed by Apical Scientific (Malaysia), and the resulting data were analyzed using the BLAST algorithm available at NCBI. Phylogenetic analysis was conducted with BioEdit and MEGA X software, employing the Fast Minimum Evolution method to generate a distance-based tree, with a maximum sequence divergence threshold of 0.75.

### Growth and metabolite production of fungal isolate under temperature stress in modified Czapek yeast broth

A modified Czapek Yeast Broth (CYB) medium, optimized by our research group, was used for metabolite production. CYB consisted of 1% glucose, 1% peptone, 0.5% KCl, 0.05% MgSO₄, and 0.001% FeSO₄·7H₂O, with the pH adjusted to 5.8 ± 0.2. To enhance fungal growth and metabolite biosynthesis, the medium was further enriched with 3% starch and 2% glucose. Fungal spores from 10 to 14 days old cultures were inoculated into the modified CYB medium and incubated in a shaking incubator at 150 rpm for 14 days under different temperatures, ranging from 30 °C to 46 °C, to induce stress responses and assess their impact on fungal metabolism. All experiments were performed in triplicate. Biomass was harvested by filtration, dried at 60 °C to constant weight, and metabolite yield was normalized to dry biomass. Salicylic acid was extracted from the culture filtrate with ethyl acetate, concentrated, and subjected to GC–MS analysis.

### Solvent-based extraction of fungal crude metabolites

After the incubation period, the fungal mycelia were homogenized using an electrical blender to release intracellular metabolites. To facilitate the separation of media components, 250 μL of 40% HCl was added to the culture. The mixture was then filtered using a vacuum filtration system, and the resulting filtrate was transferred to a separating funnel for solvent extraction. An equal volume of ethyl acetate was added to the filtrate, and the mixture was intermittently shaken for 30 min to ensure efficient metabolite partitioning. The organic layer containing the extracted metabolites was carefully collected and concentrated using a rotary evaporator set at 45 °C to remove the solvent, yielding the crude metabolite extract.

### Purification of crude metabolites using chromatographic techniques

The crude metabolite extract was purified using a series of chromatographic techniques to isolate bioactive compounds. Initially, the crude extract was mixed with a small amount of silica gel (60 mesh size) to form a free-flowing powder, ensuring even distribution when applied to the chromatographic column. A glass column was thoroughly rinsed with distilled water and 75% ethanol and allowed to dry completely before use. To prepare the stationary phase, silica gel was suspended in pure n-hexane and poured into the column to form a uniform silica bed. Once the bed was settled, the crude extract was carefully loaded onto the column, ensuring minimal disruption to the stationary phase. The column was first eluted with pure n-hexane, followed by the gradual increase of polarity by sequentially adding ethyl acetate in different volumes to facilitate differential elution of metabolites. Thin-layer chromatography (TLC) was simultaneously performed on each fraction collected from the column to monitor compound separation. The presence of distinct compounds was confirmed by observing separate bands on TLC plates under a UV lamp. To achieve further purification, preparative TLC was performed, followed by preparative high-performance liquid chromatography (HPLC) to obtain the pure compound for subsequent characterization and bioactivity assessment (Ma et al., 2016).

### Mass determination of the purified compound

The molecular mass of the purified compound was determined using gas chromatography–mass spectrometry (GC–MS). The analysis was conducted on an Agilent 7890B gas chromatograph coupled with an Agilent 5977B mass spectrometer. A 1 μL aliquot of the sample, dissolved in methanol, was injected into the system for analysis. Separation was carried out using an Agilent HP-5 ms capillary column (0.25 μm film thickness) with helium (He) as the carrier gas at a constant flow rate. The mass spectra were acquired in electron ionization (EI) mode, and data analysis was performed using Agilent Mass Hunter software, enabling the identification and mass determination of the purified compound based on its fragmentation pattern.

### Functional group determination of the purified compound

The functional groups present in the purified compound were identified using Fourier Transform Infrared (FTIR) spectroscopy. The analysis was performed on a Cary 630 FTIR spectrometer (Agilent Technologies, USA). FTIR spectroscopy measures the absorption of infrared radiation by the sample, producing a characteristic spectrum that serves as the molecular fingerprint of the compound. The sample, in powdered form, was directly analyzed to determine its functional groups and gain insights into its molecular structure. The resulting infrared absorption bands were examined and analyzed to identify specific functional groups based on their characteristic vibrational frequencies.

### NMR based structural analysis of the pure compound

For NMR analysis, 5 mg of the purified compound was accurately weighed and dissolved in 600 μL of deuterated dimethyl sulfoxide (DMSO-d_6_) to ensure complete solubility. The prepared solution was then transferred into a 5 mm NMR tube, ensuring uniform mixing and the absence of any particulate matter. The sample was carefully handled to prevent contamination or solvent evaporation before analysis. A comprehensive set of one-dimensional (^1^H NMR and ^13^C NMR) and two-dimensional (HSQC) NMR experiments were conducted using a Varian 500 MHz NMR spectrometer to determine the structural characteristics of the compound. The chemical shifts (*δ* values) were recorded in parts per million (ppm) and referenced against tetramethylsilane (TMS) as an internal standard.

### Virtual screening of the purified compound against human inflammatory protein targets

To assess the anti-inflammatory potential of the purified salicylic acid, molecular docking studies were performed against key inflammatory protein targets in the human body. The process began with the retrieval of cyclooxygenase-1 (COX-1) from *Homo sapiens*, available under PDB ID: 6Y3C (Miciaccia et al., 2021; Chokshi et al., 2021) and cyclooxygenase-2 (COX-2) from *Mus musculus*, retrieved under PDB ID: 3MDL (Vecchio and Malkowski, 2011). Additionally, other relevant targets of salicylic acid were considered, including cyclin-dependent kinase 2 (CDK2) from *Homo sapiens* under PDB ID: 5NEV (Coxon et al., 2017) and high-mobility group protein B1 (HMGB1) from *Homo sapiens* under PDB ID: 819 M. Following retrieval, all proteins were prepared for molecular docking using UCSF Chimera 1.15, where they underwent energy minimization and unwanted molecules, including water molecules and cofactors, were removed to ensure accurate docking. The salicylic acid structure was sketched using Chem Draw Ultra 16 and converted into the appropriate format for docking. Both the prepared protein targets and the salicylic acid molecule were converted into PDBQT format for docking, which was performed using PyRx 0.8, a widely used virtual screening tool. To validate the docking protocol, positive control ligands were included for each target like celecoxib for COX-2, rofecoxib for COX-1, flavopiridol for CDK2, and glycyrrhizin for HMGB1. This docking study helped evaluate the binding affinity and potential interactions between salicylic acid and key inflammatory targets, providing insights into its mechanism of action as an anti-inflammatory agent.

### Invitro anti-inflammatory activity of the pure compound

The anti-inflammatory activity of the purified salicylic acid was evaluated using the egg albumin denaturation assay. A reaction mixture was prepared using 2.8 mL of phosphate-buffered saline (PBS, pH 6.4) and 0.2 mL of hen’s egg albumin, maintained at room temperature. To assess the inhibitory effect of salicylic acid, different concentrations were prepared to achieve final concentrations of 100, 150, 200, 250, and 300 μg mL^−1^. To evaluate the combined effect of salicylic acid and ibuprofen, both compounds were mixed in equal amounts to reach the same final concentrations. Additionally, ibuprofen alone was used as a reference drug at the same concentration range. For each test sample, 2 mL of the prepared solutions were added to the reaction mixture and incubated in a water bath at 70 °C for 5 min. After cooling, the absorbance at 680 nm was recorded using a UV–visible spectrophotometer (HDTM, 2023). The percentage inhibition of protein denaturation was calculated using the following formula:


$$\begin{array}{l} \text{Percentage inhibition}\;(\%)\\ =\frac{\text{absorbance of control}-\text{absorbance of test}}{\text{absorbance of control}}\times 100 \end{array}$$

## Results

### Identification of the endophytic fungal strain

The fungal strain was identified based on its colony morphology, texture, and microscopic features. When cultured on potato dextrose agar (PDA), colonies developed with a yellow to yellow-green pigmentation, presenting a granular to floccose texture and irregular margins. The colony surface appeared loosely organized, with abundant conidiophores imparting a dusty appearance.

**Figure 1:**
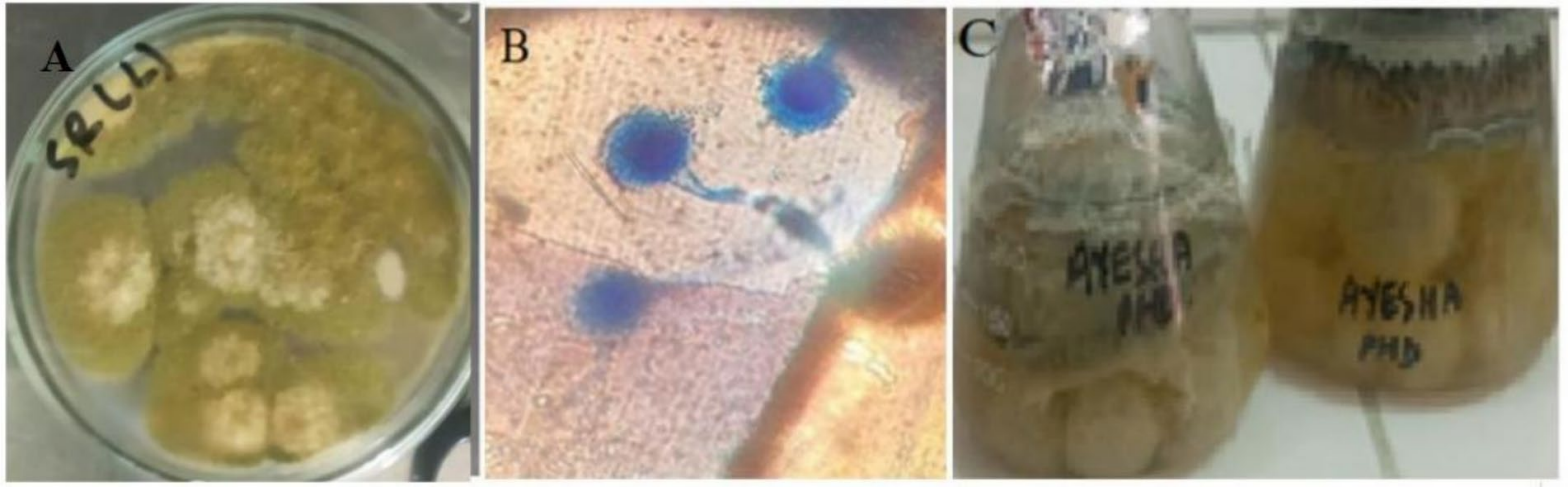
**(A)** Colony morphology of *A. flavus* grown on potato dextrose agar (PDA), exhibiting characteristic velvety-textured yellow-green colonies with radial growth. **(B)** Microscopic image of *A. flavus*, conidiophores and conidia stained with lactophenol cotton blue. **(C)** Broth culture of *A. flavus aft*er 14 days of incubation under shaking conditions, showing fungal biomass development in the modified Czapek yeast broth medium.

Microscopic examination using the slide culture method revealed hyaline, septate hyphae and conidial heads that were either uniseriate or biseriate, radiating around the vesicle. Phialides were distributed across the entire vesicle surface, which is a defining trait of *A. flavus*. The conidiophores were long, rough-walled, and non-septate, terminating in globose to subglobose vesicles. Conidia were spherical to sub-spherical, pale green, echinulate, and comparatively larger in size, consistent with the diagnostic morphology of *A. flavus* (Figure 1).

### Molecular identification of the bioactive fungal strain

**Figure 2:**
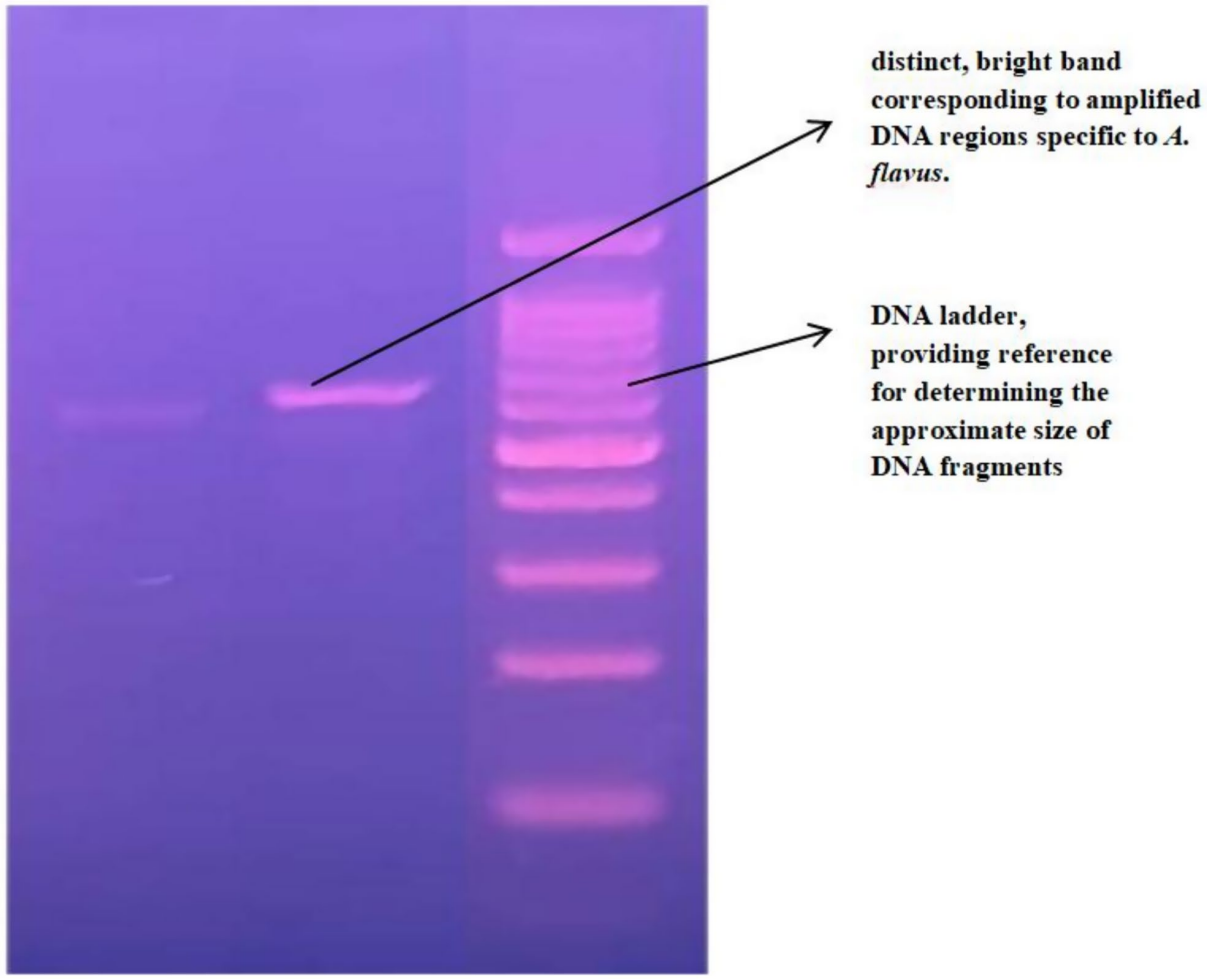
PCR amplicon of the fungal isolate visualized on a 1% agarose gel. Presents the results of agarose gel electrophoresis performed on PCR amplicons of DNA isolated from *A. flavus*. The electrophoresis was conducted using a 1% agarose gel stained with a DNA-intercalating dye and visualized under ultraviolet light. The GeneRuler 1 kb DNA ladder was used as a molecular size marker.

**Figure 3:**
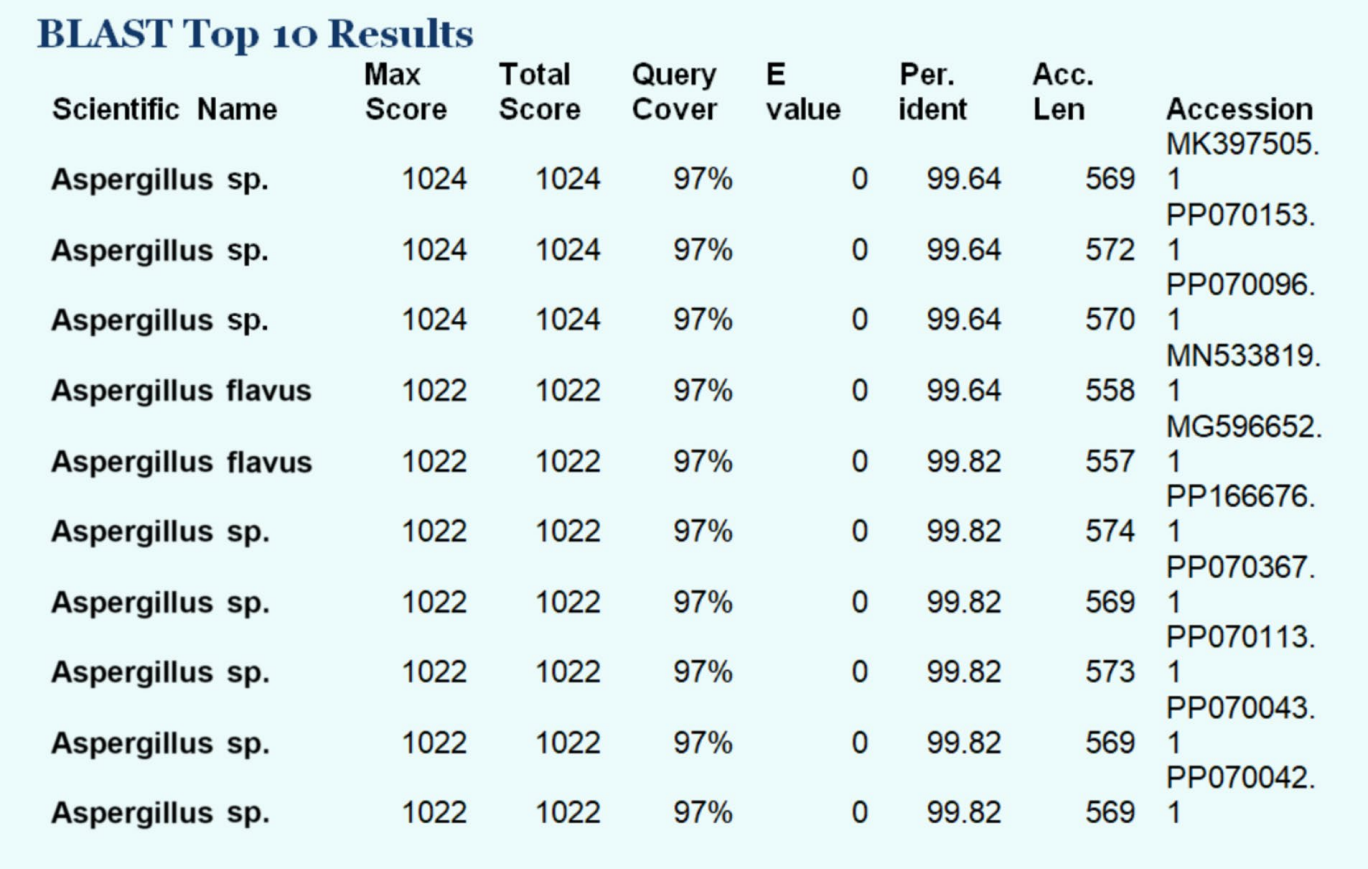
BLAST analysis of the ITS sequence of the isolated endophytic fungus. The sequence showed the highest similarity (up to 99.82% identity) with *Aspergillus* species, confirming its close taxonomic relationship to *A. flavus. Th*e top hits are listed with corresponding accession numbers, query coverage, and percentage identity.

**Figure 4:**
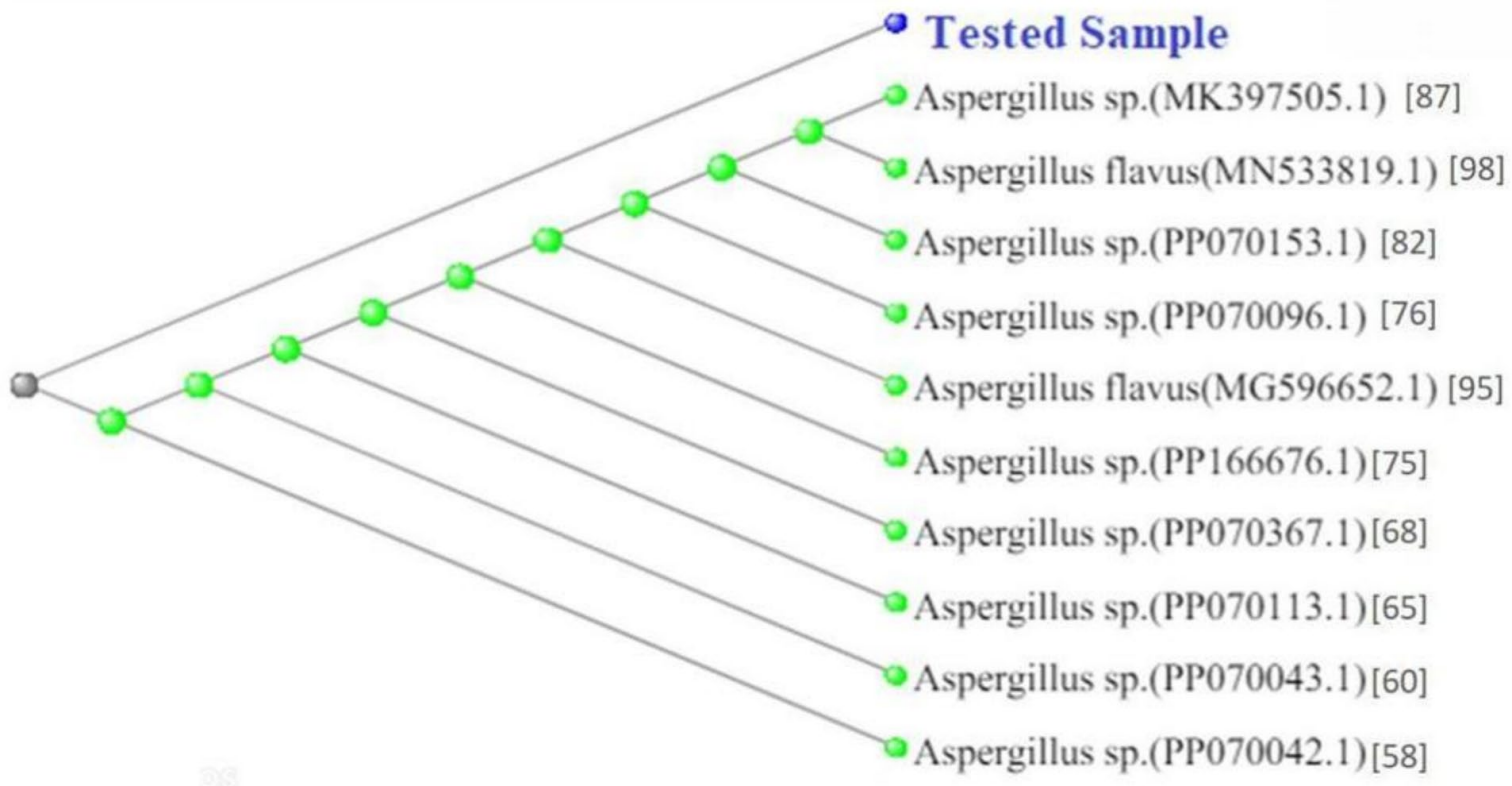
Phylogenetic tree based on ITS region sequence alignment of the tested fungal isolate. The tree shows the evolutionary relationship between the tested sample (highlighted in blue) and closely related *Aspergillus spe*cies retrieved from the NCBI GenBank database. The isolate clusters closely with *Aspergillus flavus* (*ac*cessions MN533819.1 and MG596652.1) and several *Aspergillus* sp. strains (e.g., MK397505.1, PP070153.1), confirming its taxonomic identity within the *Aspergillus gen*us. The tree was constructed using BLAST pairwise alignments, illustrating high sequence similarity and close genetic relatedness. Numbers at nodes represent bootstrap support values based on 1,000 replicates. The isolate clustered with *Aspergillus flavus* sequences, confirming its identity.

Genomic DNA was successfully extracted from the pure culture of the selected endophytic fungus using the DNeasy Mini Tissue DNA Extraction Kit (Qiagen). The quality and integrity of the extracted DNA were confirmed by electrophoresis on a 1% agarose gel, showing distinct PCR amplicons (Figure 2). The internal transcribed spacer (ITS) region was sequenced and subjected to BLAST analysis, which revealed a 99.82% identity with *Aspergillus flavus*, confirming its molecular identity (Gene bank accession number MN533819.1) (Figure 3). A phylogenetic tree was constructed using MEGA X to further validate the evolutionary relationship of the isolate, bootstrap test with 1,000 replicates was conducted. The resulting tree showed that our isolate grouped closely with *Aspergillus flavus* reference sequences, supported by high bootstrap values (95–98%), thereby confirming its molecular identity as shown in Figure 4.

### Purification and characterization of salicylic acid

**Figure 5:**
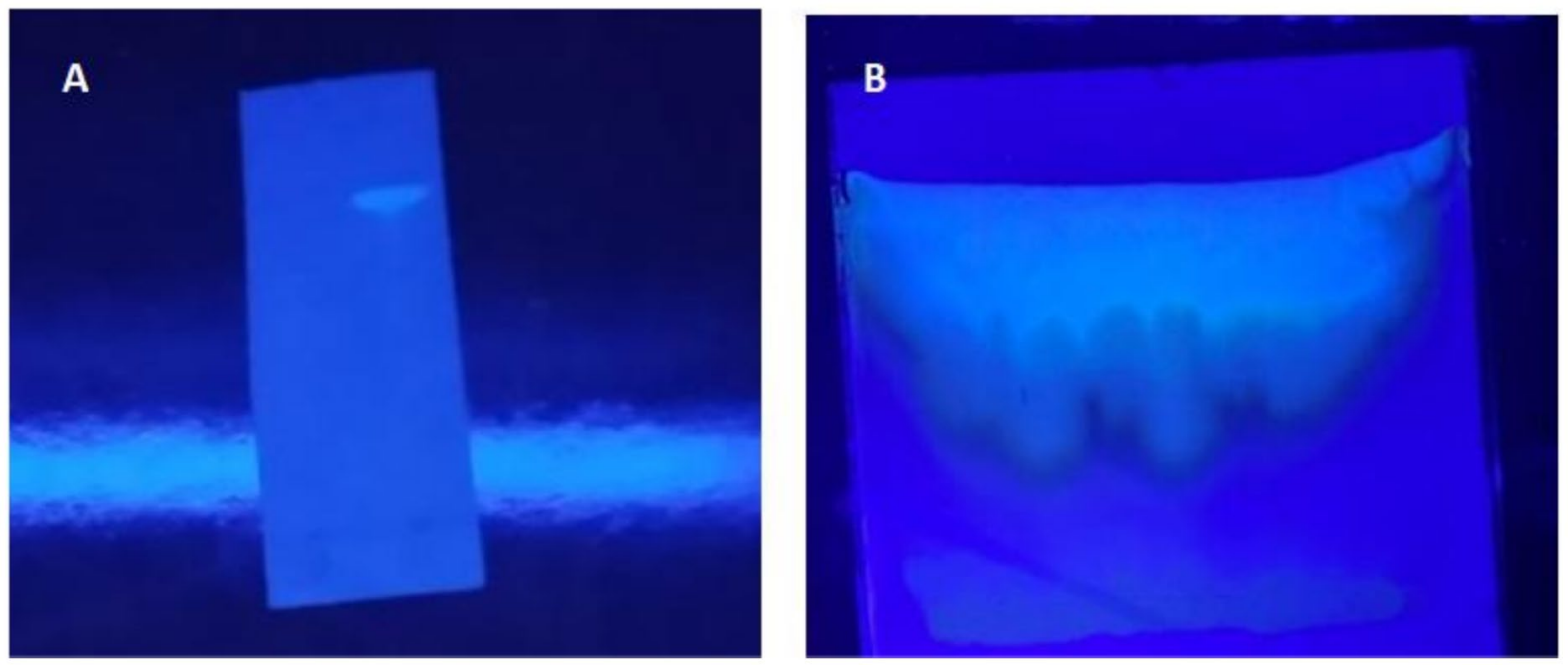
**(A)** Thin-layer chromatography (TLC) analysis of purified salicylic acid under UV light, showing a single sharp fluorescent band, confirming its purity. The separation was achieved using a solvent system of 45% ethyl acetate and 55% *n*-hexane. **(B)** Preparative TLC.

**Figure 6:**
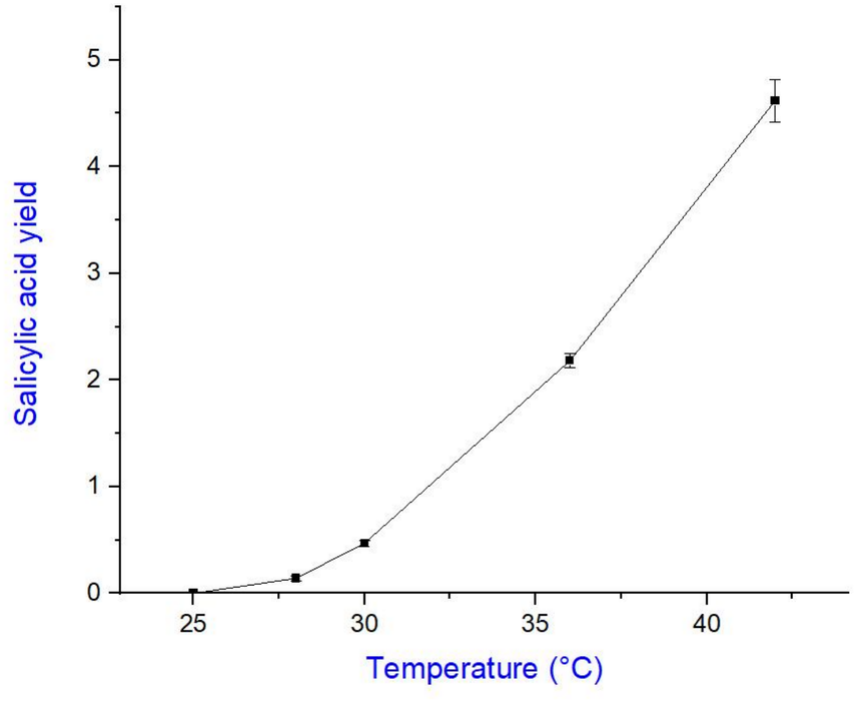
Effect of temperature stress on salicylic acid production by *Aspergillus flavus*. Data points represent mean ± SD. The optimum growth temperature (28 °C) served as the control. Production increased significantly at 30 °C, 36 °C, and peaked at 42 °C.

**Figure 7:**
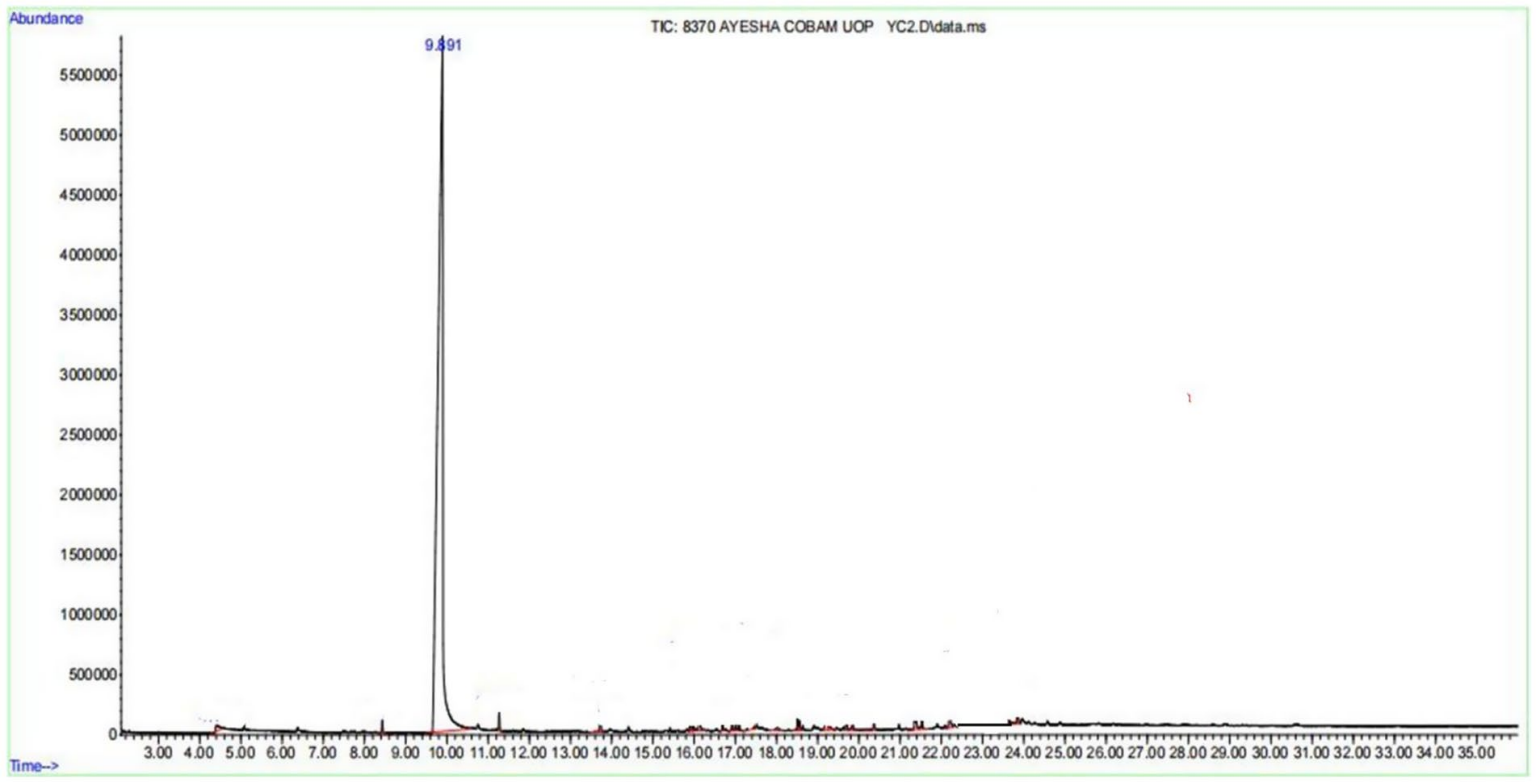
GC–MS chromatogram (TIC) of the purified salicylic acid showing a single sharp peak at a retention time of 9.8 min.

**Figure 8:**
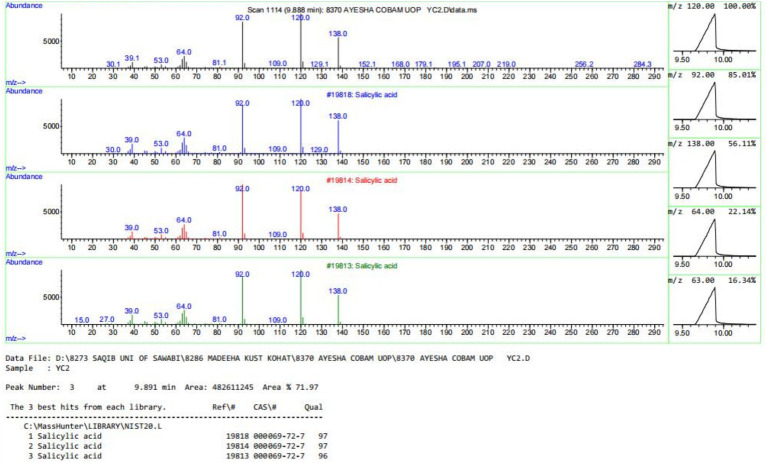
Mass spectrum of the purified salicylic acid obtained from the Agilent GC–MS system showing a molecular ion peak at m/z 138, corresponding to the molecular weight of salicylic acid, along with characteristic fragment ions supporting its structural confirmation. The total ion chromatogram (TIC) represents the separation profile, where the Y-axis indicates ion abundance and the X-axis represents retention time, confirming the purity of the compound.

**Figure 9:**
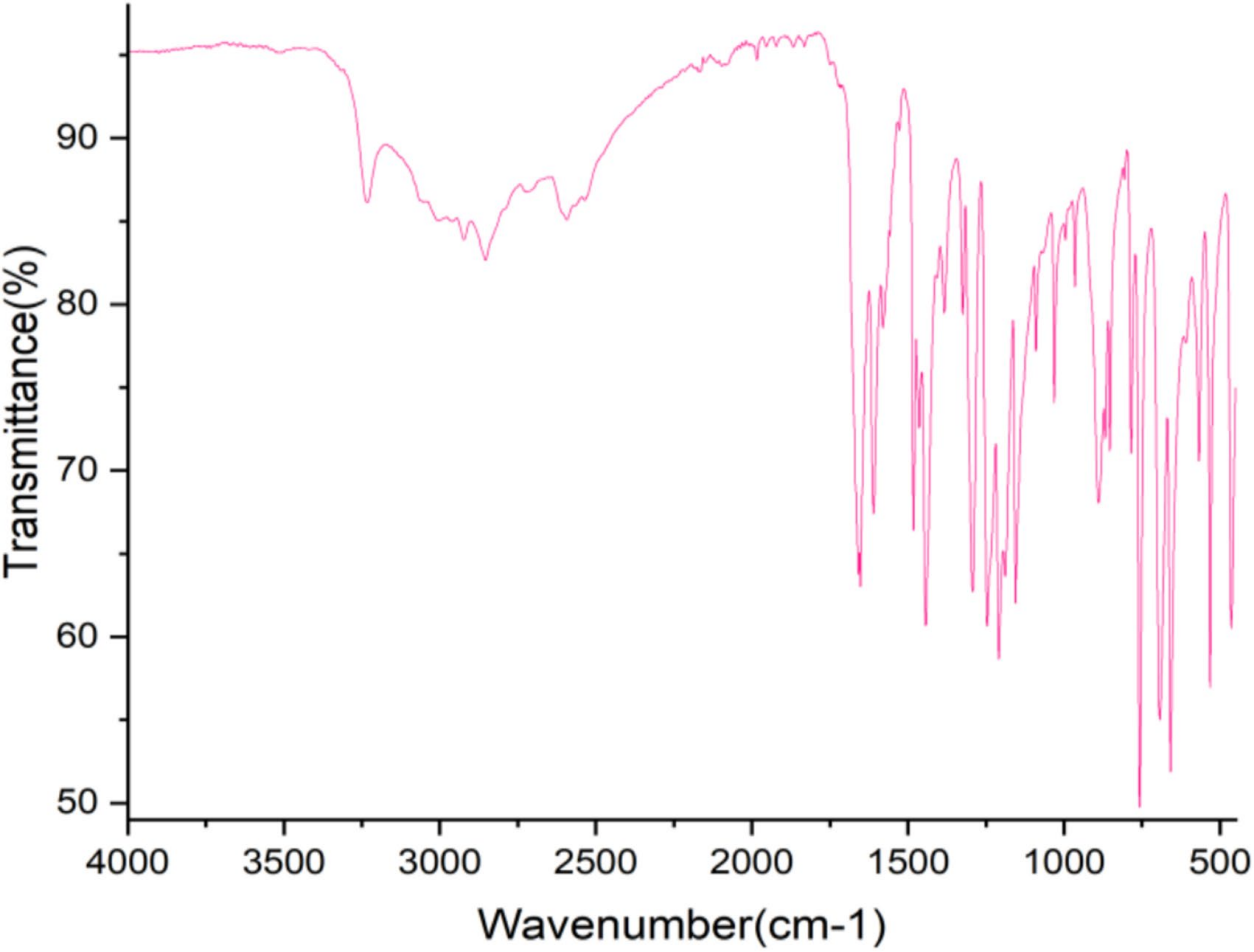
FTIR spectrum of the purified salicylic acid, showing characteristic absorption bands corresponding to functional groups.

The purification of salicylic acid was carried out using column chromatography, employing a gradient solvent system of ethyl acetate and n-hexane in varying ratios to achieve differential polarity for effective separation. Throughout the purification process, thin-layer chromatography (TLC) was routinely performed to monitor the separation of compounds. Under UV light, salicylic acid appeared as a single sharp band, indicating its purity (Figure 5). The optimal solvent system for purification was determined to be 45% ethyl acetate and 55% n-hexane, which yielded the best separation of salicylic acid from other metabolites. The amount of purified salicylic acid varied with incubation temperature, indicating a temperature-dependent biosynthesis. At the optimum growth temperature (28 °C), Salicylic acid yield was 0.14 ± 0.02 mg/200 mL medium. A moderate increase was observed at 30 °C (0.47 ± 0.03 mg), representing a 3.4 fold rise compared to the control (*p* ≈ 0.01). Production peaked at 42 °C (4.62 ± 0.20 mg), corresponding to a 33 fold increase (*p* < 0.001), while 36 °C also showed a strong induction (2.18 ± 0.07 mg, 15.6-fold, *p* < 0.001). No detectable Salicylic acid was produced at 25 °C. These findings suggest that elevated temperature enhances salicylic acid production, with 42 °C being the most favorable condition for its biosynthesis using *A. flavus*. The dataset is tabulated in Table 1 and graphically represented in Figure 6. The purified compound was further analyzed for its structural confirmation and purity using gas chromatography–mass spectrometry (GC–MS) and Fourier-transform infrared spectroscopy (FTIR). The GC–MS chromatogram exhibited a single sharp peak at a retention time of 9.88 min, indicating a pure compound (Figure 7). The mass spectrum revealed a molecular ion peak at m/z 138, which corresponds precisely to the molecular weight of salicylic acid (Figure 8). Additionally, the fragmentation pattern was consistent with the expected structure, with characteristic fragment ions observed at m/z 120, 92, and 64, supporting the presence of functional moieties associated with salicylic acid. Further characterization was performed using FTIR spectroscopy, which provided insights into the functional groups present in the purified compound. The FTIR spectrum exhibited a broad absorption band between 2,500–3,400 cm−1, characteristic of the hydroxyl (-OH) group attached to the benzene ring. A sharp peak around 1700 cm−1 confirmed the presence of a carbonyl (C=O) group, indicative of the carboxyl functional group in salicylic acid. Additional absorption bands between 1,200–1,300 cm−1 corresponded to C-O stretching vibrations in both the phenolic and carboxylic acid groups, while bands in the 700–900 cm−1 range were attributed to the aromatic C-H bending vibrations. The combined GC–MS and FTIR analyses provide strong evidence for the identification of the purified compound as salicylic acid, confirming the presence of hydroxyl and carboxyl functional groups while ensuring its structural integrity and high purity (Figure 9).

**Table 1:** Effect of temperature stress on salicylic acid production by endophytic *Aspergillus flavus* (1,000 mL media, 14 days incubation).

Sr no	Temp(°C)	Salicylic acid (mg/200 ml media)	Mean ± SD	Fold vs. 28 °C	*Post hoc p*-value	Significance
1	25	0	0	–	–	–
2	28	0.12, 0.16, 0.14	0.14 ± 0.02	1.00	–	Control
3	30	0.45, 0.50, 0.46	0.47 ± 0.03	3.36	*p* ≈ 0.01	Significant
4	36	2.10, 2.20, 2.24	2.18 ± 0.07	15.57	*p* < 0.001	Highly significant
5	42	4.40, 4.80, 4.66	4.62 ± 0.20	33.0	*p* < 0.001	Highly significant

Data are presented as mean ± SD. Fold changes are calculated relative to the optimum growth temperature (28 °C, control). Statistical significance was determined using *post hoc t*-test.

### Nuclear magnetic resonance (NMR) based description of the isolated compound

**Figure 10:**
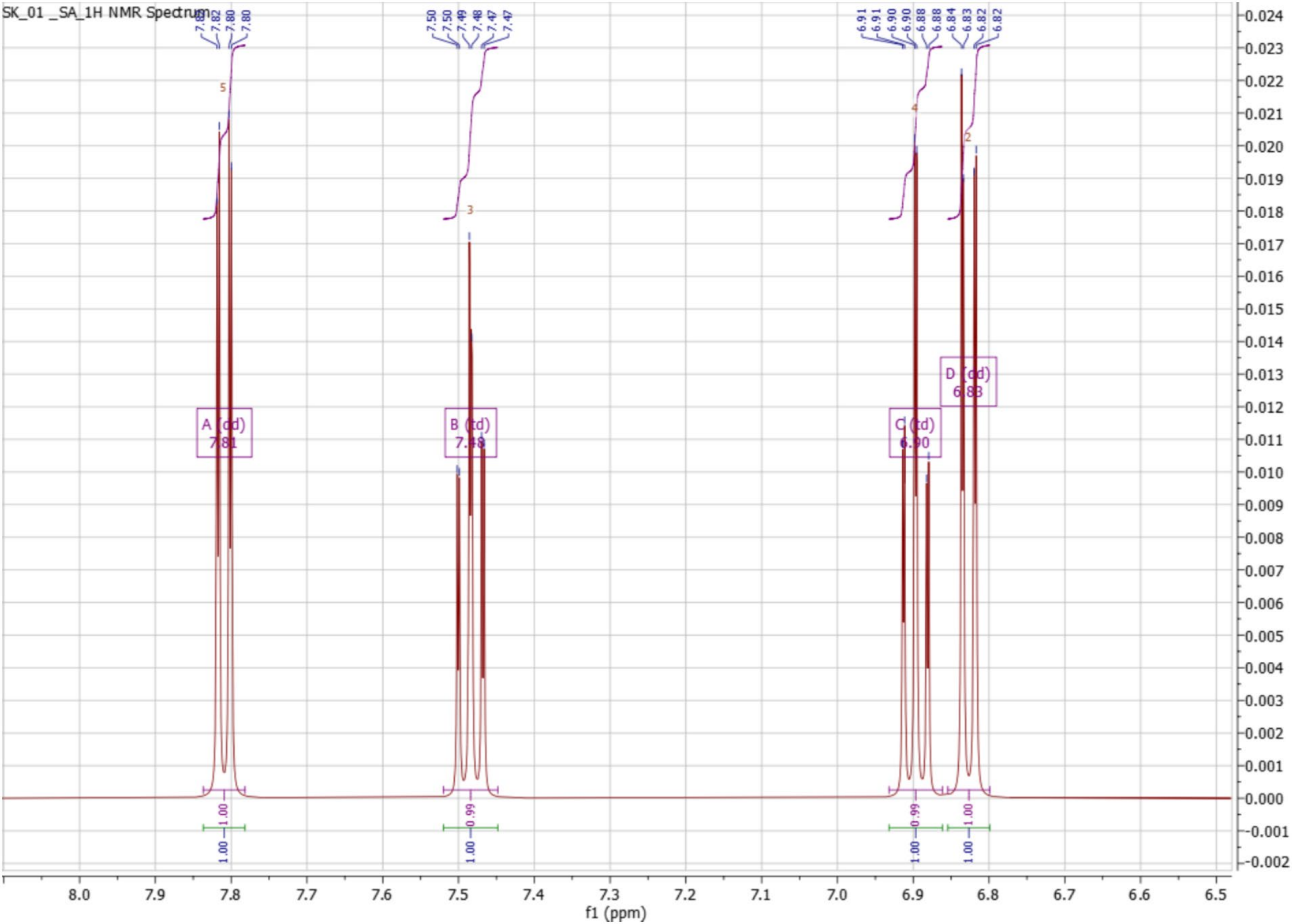
Proton NMR of salicylic acid.

**Figure 11:**
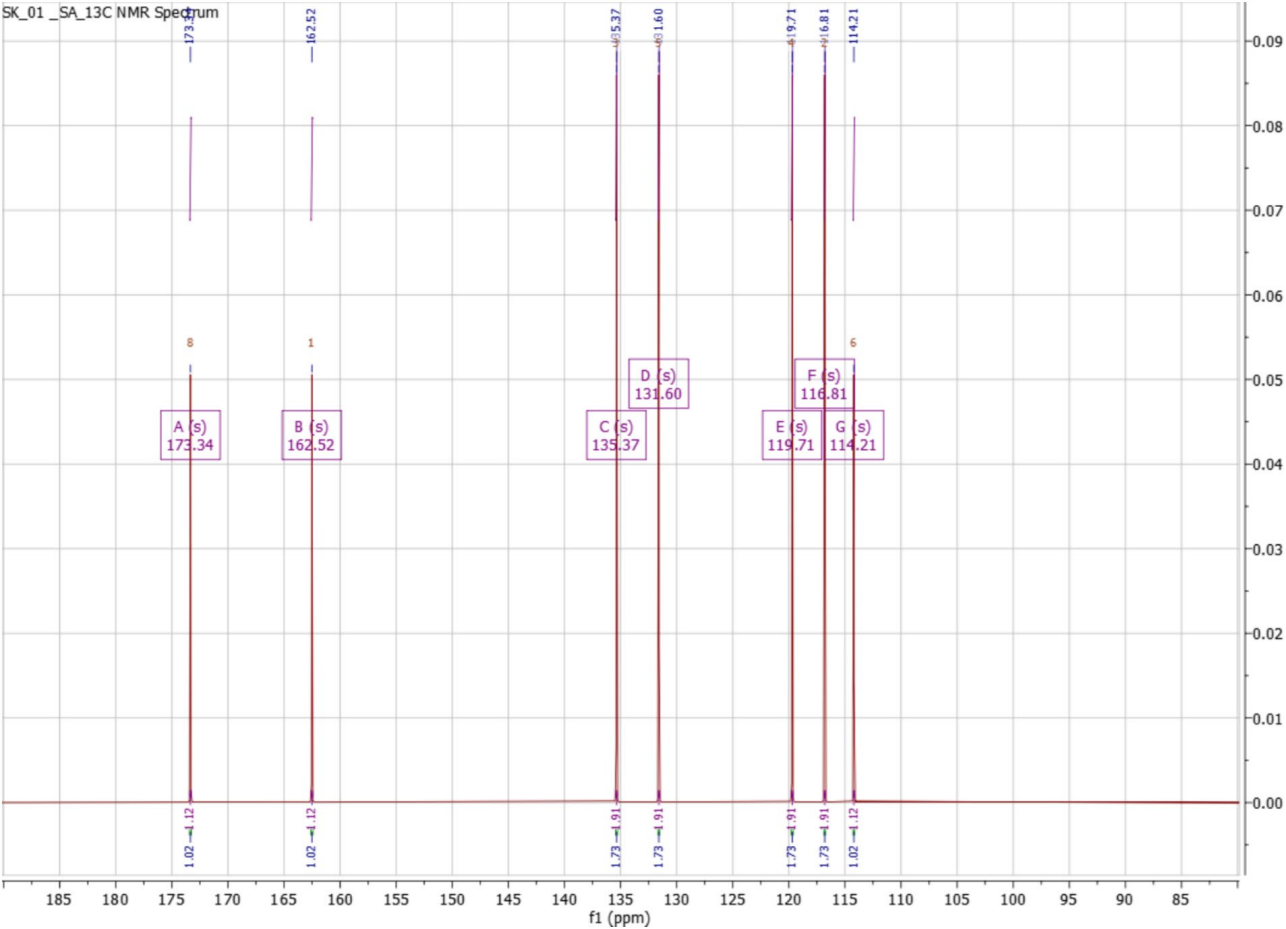
Carbon 13 NMR of salicylic acid.

The ^1^H NMR spectrum of the purified compound exhibited distinct signals in the aromatic region, indicative of a benzene ring with specific substitutions. The presence of doublets around *δ* 7.80 ppm and δ 7.49 ppm suggests an aromatic system with characteristic coupling patterns, likely corresponding to protons in a meta or para configuration. Additionally, signals appearing at δ 6.90–6.80 ppm further support the presence of aromatic protons, likely positioned ortho to the functional groups. The splitting pattern observed in the spectrum is consistent with a disubstituted benzene ring, which aligns well with the expected structure of salicylic acid. The absence of any aliphatic signals further reinforces this structural assignment (Figure 10). The ^13^C NMR spectrum provides further insight into the molecular structure, with a strong deshielded signal at δ 173.34 ppm, characteristic of a carboxyl (-COOH) carbon, confirming the presence of a carboxylic acid functional group. A resonance at δ 162.52 ppm corresponds to a hydroxyl (-OH) bearing carbon, supporting the hydroxyl-substituted aromatic system. Additional peaks between δ 135.37 and 114.21 ppm correspond to the sp^2^-hybridized carbons of the benzene ring, with distinct chemical shifts indicating specific substitutions at different positions. These assignments align with the expected chemical environment of salicylic acid, where hydroxyl and carboxyl groups are present on an aromatic system (Figure 11).

**Figure 12:**
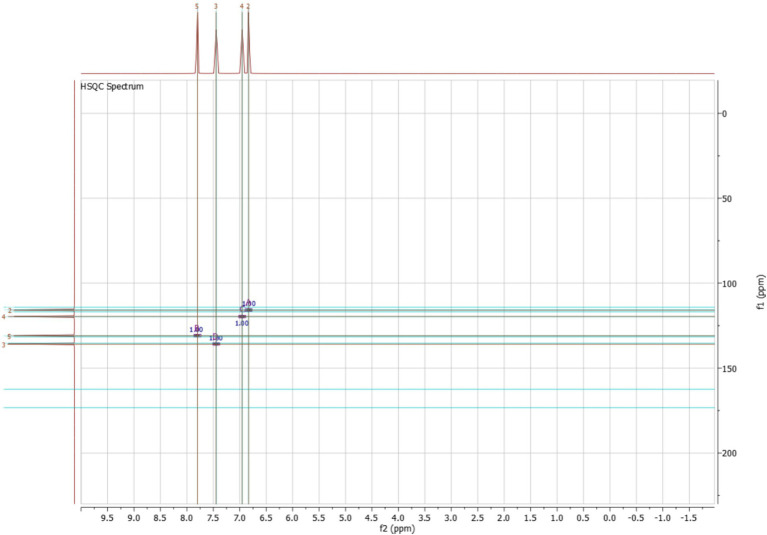
HSQC NMR of salicylic acid.

**Figure 13:**
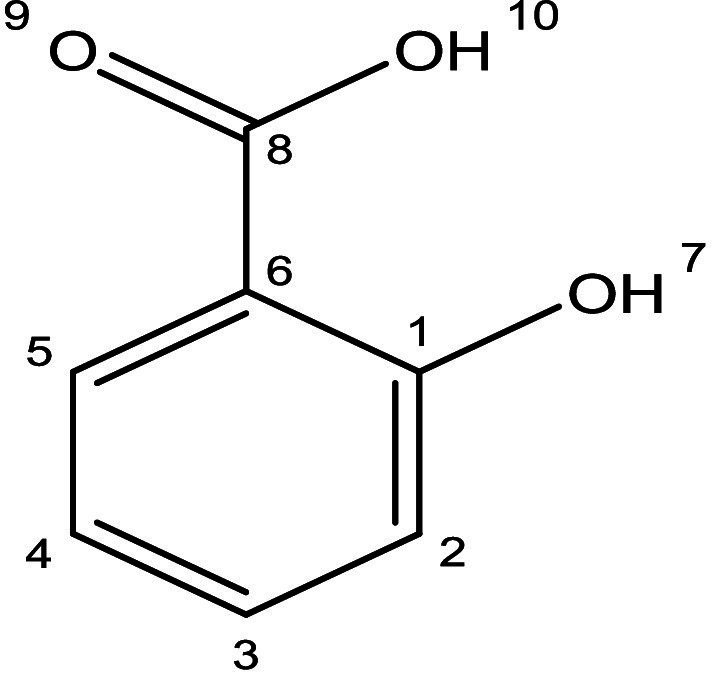
Elucidated structure of salicylic acid.

The HSQC spectrum further supports these findings by establishing direct proton-carbon correlations. The observed signals confirm the expected connectivity of the aromatic protons with their respective carbon environments, reinforcing the substitution pattern on the benzene ring. The absence of additional unexpected correlations suggests that the compound is structurally pure and corresponds well with salicylic acid. Overall, the combined ^1^H, ^13^C and HSQC NMR data strongly support the structural characterization of the purified compound as salicylic acid, confirming the presence of hydroxyl and carboxyl functional groups on a benzene ring. The well-defined splitting patterns, characteristic chemical shifts, and solvent compatibility further reinforce the reliability of this structural assignment (Figure 12). Based on the findings, the deduced structure corresponds to salicylic acid, as illustrated in Figure 13.

### Molecular docking studies

**Figure 14:**
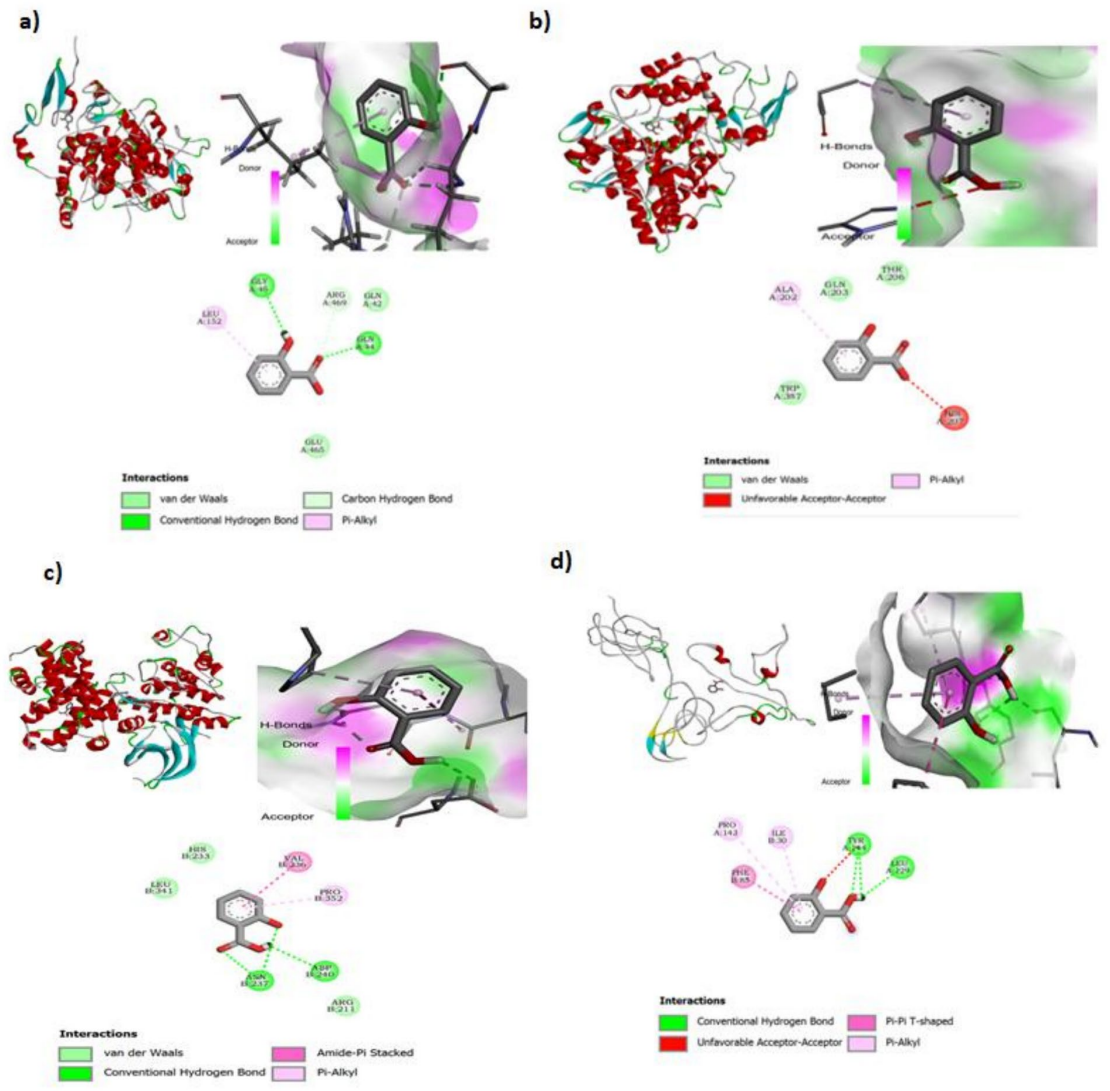
**(a)** Molecular docking analysis of salicylic acid with cyclooxygenase-1 (COX-1), showing its binding conformation within the active site. The interaction map highlights key hydrogen bonds, van der Waals forces, and pi-alkyl interactions with amino acid residues, suggesting a potential inhibitory effect on COX-1. **(b)** Molecular docking analysis of salicylic acid with cyclooxygenase-2 (COX-2), illustrating its binding conformation within the enzyme’s active site. The interaction map highlights key molecular interactions, including hydrogen bonding, van der Waals forces, pi-alkyl interactions, and unfavorable acceptor-acceptor interactions, suggesting its potential role in COX-2 inhibition. **(c)** Molecular docking analysis of salicylic acid with cyclin-dependent kinase 2 (CDK2), illustrating its binding conformation within the enzyme’s active site. The interaction map highlights key molecular interactions, including conventional hydrogen bonding, van der Waals forces, amide-pi stacking, and pi-alkyl interactions, suggesting a potential inhibitory effect on CDK2. **(d)** Molecular docking analysis of salicylic acid with high-mobility group protein B1 (HMGB1), illustrating its binding conformation within the active site. The interaction map highlights key molecular interactions, including conventional hydrogen bonding, pi-pi T-shaped interactions, pi-alkyl interactions, and unfavorable acceptor-acceptor interactions, suggesting a potential role of salicylic acid in modulating HMGB1-associated inflammatory pathways.

To better understand the anti-inflammatory potential of salicylic acid, molecular docking was performed with key inflammatory protein targets in humans. These included cyclooxygenase-1 (COX-1), cyclooxygenase-2 (COX-2), cyclin-dependent kinase 2 (CDK2), and high-mobility group protein B1 (HMGB1). The docking scores obtained were −6.0 for COX-1, −5.5 for COX-2, −6.1 for CDK2, and −4.9 for HMGB1, with lower docking scores indicating stronger binding affinity. For comparison, positive control ligands showed substantially stronger affinities, celecoxib with COX-2 (−9.5 kcal/mol), rofecoxib with COX-1 (−8.0 kcal/mol), flavopiridol with CDK2 (−8.5 kcal/mol), and glycyrrhizin with HMGB1 (−8.0 kcal/mol). Among the targets, salicylic acid exhibited the strongest interaction with CDK2, as reflected by its lowest docking score of −6.1. This suggests that CDK2 could be a key molecular target for its anti-inflammatory activity. The relatively high binding affinities observed for COX-1 and COX-2 also indicate that salicylic acid may influence inflammatory pathways involving these enzymes. These docking results provide valuable insights into the potential mechanism of salicylic acid as an anti-inflammatory agent, suggesting that its activity may be mediated through interactions with multiple inflammation-associated proteins. The detailed docking results are illustrated in Table 2 and Figure 14.

**Table 2:** Molecular docking scores (kcal/mol) of salicylic acid and positive control ligands with key inflammatory protein targets.

SR no	Protein target	Salicylic acid docking score (kcal/mol)	Positive control ligand	Control docking score (kcal/mol)
1	COX-1	−6.0	Rofecoxib	−9.5
2	COX-2	−5.5	Celecoxib	−8.0
3	CDK2	−6.1	Flavopirido	−8.5
4	HMGB1	−4.9	Glycyrrhizin	−8.0

### *In vitro* anti-inflammatory activity

The anti-inflammatory activity of purified salicylic acid from *A. flavus*, ibuprofen, and their combination was evaluated using the egg albumin denaturation assay to assess their inhibitory effect on protein denaturation. All experiments were performed in triplicate, and results are expressed as Mean ± SD. Statistical analysis was conducted using one-way ANOVA followed by Tukey’s post-hoc test in IBM SPSS Statistics.

The results indicated that ibuprofen exhibited a higher inhibition rate compared to fungal derived salicylic acid at all tested concentrations. However, the combination of salicylic acid and ibuprofen demonstrated the highest inhibition, with a maximum inhibition observed at 300 μg mL^−1^. Furthermore, the inhibition rates increased progressively with increasing concentration, indicating a dose-dependent response. ANOVA revealed highly significant differences among treatments (*p* < 0.0001), and Tukey’s *post hoc* test confirmed that the combination exhibited significantly higher inhibition compared to salicylic acid (*p* < 0.001) and ibuprofen (*p* < 0.001) alone, while ibuprofen was also significantly stronger than salicylic acid (*p* < 0.001).

**Figure 15:**
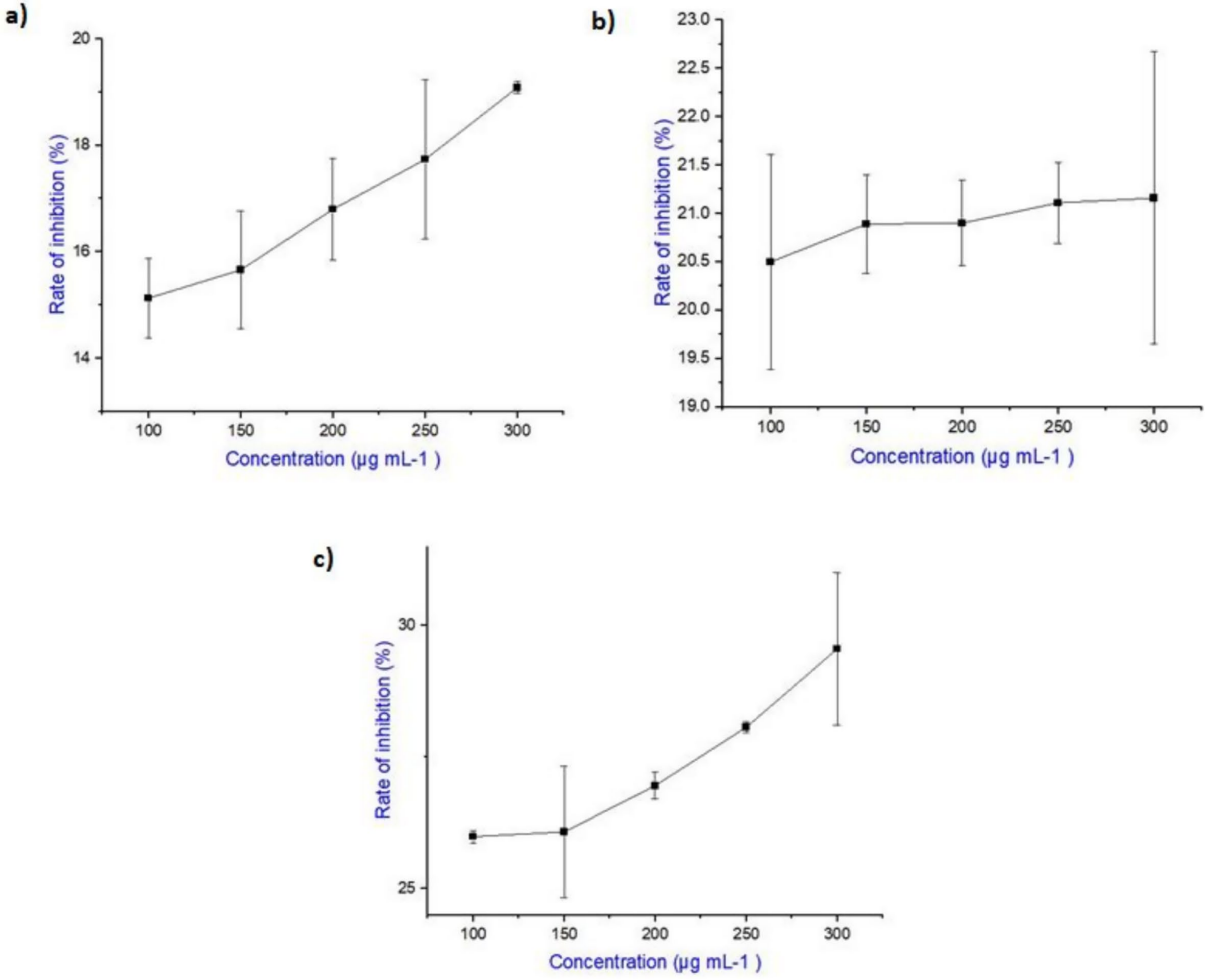
**(a)** Dose-dependent inhibition of protein denaturation by salicylic acid. The figure illustrates the inhibitory effect of salicylic acid on protein denaturation at varying concentrations (100–300 μg mL^−1^). The results indicate a progressive increase in inhibition with increasing concentration, demonstrating a dose-dependent response. **(b)** Dose-dependent inhibition of protein denaturation by ibuprofen. The figure represents the inhibitory effect of ibuprofen on protein denaturation across different concentrations (100–300 μg mL^1^). The results show a progressive increase in inhibition with increasing concentration, highlighting its potent anti-inflammatory activity. **(c)** Inhibition of protein denaturation by the combination of salicylic acid and ibuprofen. The figure illustrates the dose-dependent inhibitory effect of the combined treatment of salicylic acid and ibuprofen on protein denaturation. The results demonstrate a progressive increase in inhibition with increasing concentration (100–300 μg mL^−1^), with the combination exhibiting a higher inhibitory effect compared to either compound alone, suggesting an enhanced combined anti-inflammatory potential of salicylic acid and ibuprofen. Error bars shown in the graphs represent standard deviations (SD) from triplicate experiments.

The detailed inhibitory effects of salicylic acid, ibuprofen and their combination are presented in Table 3 and illustrated in Figure 15. These findings suggest that salicylic acid enhances the anti-inflammatory potential of ibuprofen, making their combination a promising natural adjunct for anti-inflammatory therapy.

**Table 3:** *In vitro* anti-inflammatory activity of salicylic acid, ibuprofen and their combination against protein denaturation.

Sr no	Concentration (μg mL^−1^)	Salicylic acid mean ± SD	Ibuprofen mean ± SD	Salicylic acid and ibuprofen combined effect mean ± SD	*P* value	Significance
1	100	15.13 ± 0.75	20.50 ± 1.11	25.99 ± 0.12	<0.0001	Significant
2	150	15.66 ± 1.11	20.89 ± 0.51	26.08 ± 1.25	<0.0001	Significant
3	200	16.80 ± 0.95	20.90 ± 0.44	26.96 ± 0.25	<0.0001	Significant
4	250	17.74 ± 1.50	21.11 ± 0.42	28.07 ± 0.11	<0.0001	Significant
5	300	19.09 ± 0.11	21.16 ± 1.51	29.56 ± 1.45	<0.0001	Significant

Values are expressed as mean ± SD (*n* = 3). One way ANOVA revealed highly significant differences among treatments (*p* < 0.0001).

## Discussion

Salicylic acid and its derivatives play a crucial role in combating inflammation, particularly as inflammatory diseases continue to rise globally. Traditionally, plants have been the primary source of salicylic acid; however, large-scale extraction from plants raises ecological concerns, necessitating the exploration of alternative sources. In this study, *A. flavus* was selected as a microbial model for salicylic acid production due to its well-documented ability to synthesize a wide range of secondary metabolites (Frisvad et al., 2009). *R. officinalis* is recognized for its anti inflammatory properties (Gonçalves et al., 2022). In this study, we isolated *A. flavus* as an endophyte from rosemary and investigated its potential as an independent microbial producer of salicylic acid.

Salicylic acid production is known to be enhanced under stress conditions, which guided our approach of leveraging stress-induced responses to optimize its biosynthesis. By subjecting *A. flavus* to controlled temperature variations, we aimed to enhance its salicylic acid production potential. Our findings revealed a significant increase in salicylic acid yield at 42 °C, compared to lower yields at 30 °C and 25 °C, highlighting the impact of temperature stress on metabolic pathways. The observed temperature-dependent increase in salicylic acid yield may reflect transcriptional reprogramming of metabolic pathways under stress, as reported in transcriptomic studies where heat stress activates secondary metabolite biosynthetic genes. These results emphasize the feasibility of using *A. flavus* as a scalable microbial source for salicylic acid production. However, it is important to note that *Aspergillus flavus* is a known producer of aflatoxins and other toxic metabolites. Although our study focused specifically on the isolation and purification of salicylic acid rather than crude extracts, comprehensive toxin profiling and aflatoxin screening remain mandatory steps before proposing pharmaceutical relevance. This safety aspect has been highlighted to ensure transparency and to clarify the clinical relevance of microbial derived salicylic acid.

The purified salicylic acid obtained from *A. flavus* holds great potential for chemical modification, offering opportunities for the development of various derivatives with broad clinical applications (Sambyal and Singh, 2021). Acetyl2-Hydroxybenzoic acid (aspirin) is widely known for its anti-inflammatory, analgesic, and antipyretic properties (Chen et al., 2018), while methyl 2- Hydroxylbenzoate is commonly used as a topical analgesic and anti-inflammatory agent. Sodium 2-Hydroxybenzoate is employed in the treatment of rheumatoid arthritis (Amann and Peskar, 2002), and mesalazine is a key therapeutic agent in managing inflammatory bowel diseases such as ulcerative colitis and Crohn’s disease (de Barros Cardoso et al., 2019). The ability to generate salicylic acid from a microbial source rather than plants reinforces the potential for large-scale production, reducing ecological impact while supporting pharmaceutical applications (Sambyal and Singh, 2021).

While salicylic acid and its derivatives are often associated with the inhibition of inflammatory targets such as cyclooxygenase-1 (COX-1), cyclooxygenase-2 (COX-2), cyclin-dependent kinase 2 (CDK2), and high-mobility group protein B1 (HMGB1), the exact mechanism of action remains only partially understood. Our molecular docking analysis revealed that salicylic acid exhibited the highest binding affinity toward CDK2, suggesting that CDK2 inhibition may represent a key mechanism underlying its anti-inflammatory effects. This interaction was notably stronger than those observed with COX-1, COX-2, and HMGB1, further supporting the hypothesis that CDK2 plays a central role in the biological activity of salicylic acid.

CDK2, a member of the cyclin-dependent kinase family, is essential for cell cycle regulation. Salicylic acid appears to act as a CDK2 inhibitor, preventing the phosphorylation of retinoblastoma protein, a key regulator of cell cycle progression. By blocking CDK2 activity, salicylic acid induces cell cycle arrest, potentially slowing or halting the proliferation of cells. Given the critical role of CDK2 in cancer progression, this mechanism suggests that salicylic acid could have therapeutic potential beyond inflammation, particularly in targeting cancer pathways. The promising molecular docking results against CDK2 warrant further exploration into the role of salicylic acid in cancer treatment, highlighting its potential application in novel therapeutic strategies.

Furthermore, our *in vitro* anti-inflammatory study demonstrated a clear correlation between the concentration of salicylic acid, ibuprofen, and their combination with the inhibition of protein denaturation. While both salicylic acid and ibuprofen independently exhibited significant anti-inflammatory effects, their combined effect, led to a higher inhibition rate than either agent alone. This suggests that the co-administration of salicylic acid and ibuprofen could enhance therapeutic efficacy in conditions where protein denaturation plays a pivotal role in inflammation. These findings highlight the potential benefits of combining these compounds for the development of more effective anti-inflammatory treatments. Our study provides a preliminary basis for exploring the combined anti-inflammatory effects of salicylic acid and ibuprofen. While our current findings highlight this potential, further work is needed to advance the analysis. Future studies should include cell-based assay, in-vitro cytokine profiling (TNF-*α*, IL-6, IL-1β) and COX enzyme inhibition assays to validate and expand these observations. However, the present study emphasizes the feasibility of microbial derived salicylic acid as an alternative to industrial synthesis, improving salicylic acid yields by optimizing temperature and supports its potential as an anti-inflammatory agent through CDK2 inhibition using molecular docking, preliminary invitro antiinflammatory assays and also demonstrates its combined effect with ibuprofen, offering new insights into its broader therapeutic applications.

## Conclusion

The findings of this study demonstrate that *A. flavus* can serve as a practical microbial source for salicylic acid production, providing a sustainable and biologically driven alternative to conventional chemical synthesis. Microbial biosynthesis offers eco-friendly production and opens new biotechnological avenues for salicylic acid applications. The use of temperature stress as a strategy to enhance salicylic acid biosynthesis was successful, with 42 °C identified as the optimal condition for maximizing yield. Molecular docking analysis suggests that CDK2 may be a key target of salicylic acid, indicating a possible alternative anti-inflammatory mechanism beyond COX inhibition. The observed strong binding affinity to CDK2 suggests that salicylic acid may play a role in cell cycle regulation, which could be further explored for its implications in cancer therapy.

Furthermore, the *in vitro* anti-inflammatory assay confirmed the effectiveness of salicylic acid in inhibiting protein denaturation, with its combination with ibuprofen resulting in a greater inhibitory effect than either compound alone. This suggests that combining salicylic acid with conventional NSAIDs could enhance anti-inflammatory efficacy, potentially reducing required dosages and associated side effects. This study concludes that fungal-derived salicylic acid has strong anti-inflammatory potential, may act as a CDK2 inhibitor for therapeutic use and works synergistically with ibuprofen, highlighting its potential for further pharmacological research. However, given that *A. flavus* is a known producer of aflatoxins and other toxic metabolites, future work must include comprehensive toxin profiling and aflatoxin screening to ensure safety and clinical applicability of *A. flavus* derived compounds.

## Data Availability

The datasets presented in this study can be found in online repositories. The names of the repository/repositories and accession number(s) can be found in the article/supplementary material.

## References

[ref1] Akhtar M. A. (2022). Anti-inflammatory medicinal plants of Bangladesh—a pharmacological evaluation. Front. Pharmacol. 13:809324. doi: 10.3389/fphar.2022.809324, 35401207 PMC8987533

[ref2] Amann R. Peskar B. A. (2002). Anti-inflammatory effects of aspirin and sodium salicylate. Eur. J. Pharmacol. 447, 1–9. doi: 10.1016/S0014-2999(02)01828-9 12106797

[ref3] Ambrose K. V. Tian Z. Wang Y. Smith J. Zylstra G. Huang B. . (2015). Functional characterization of salicylate hydroxylase from the fungal endophyte Epichloë festucae. Sci. Rep. 5:10939. doi: 10.1038/srep10939, 26055188 PMC4460724

[ref4] Chen Y.-C. Qiang G.-F. Du G.-H. (2018). “Salicylic acid,” in Natural Small Molecule Drugs from Plants, eds. Du G.-H. Chen Y.-C. Qiang G.-F. (Singapore: Springer), 455–459.

[ref5] Chokshi R. Bennett O. Zhelay T. Kozak J. A. (2021). NSAIDs naproxen, ibuprofen, salicylate, and aspirin inhibit TRPM7 channels by cytosolic acidification. Front. Physiol. 12:727549. doi: 10.3389/fphys.2021.727549, 34733174 PMC8558630

[ref6] Cipolla G. Crema F. Sacco S. Moro E. De Ponti F. Frigo G. (2002). Nonsteroidal anti-inflammatory drugs and inflammatory bowel disease: current perspectives. Pharmacol. Res. 46, 1–6. doi: 10.1016/S1043-6618(02)00033-6 12208114

[ref7] Coxon C. R. Anscombe E. Harnor S. J. Martin M. P. Carbain B. Golding B. T. . (2017). Cyclin-dependent kinase (CDK) inhibitors: structure–activity relationships and insights into the CDK-2 selectivity of 6-substituted 2-arylaminopurines. J. Med. Chem. 60, 1746–1767. doi: 10.1021/acs.jmedchem.6b01254, 28005359 PMC6111440

[ref8] de Barros Cardoso C. R. Castro Habka A. Pinzan C. F. Oliveira C. N. S. da Silva J. L. Duarte-Silva M. (2019). “Traditional drugs: mechanisms of immunosuppressor and corticosteroid therapies for inflammatory bowel diseases,” in Biological Therapy for Inflammatory Bowel Disease, ed. Quraishi A. A. (London: IntechOpen).

[ref9] Dinarello C. A. (2010). Anti-inflammatory agents: present and future. Cell 140, 935–950. doi: 10.1016/j.cell.2010.02.043, 20303881 PMC3752337

[ref10] Ekinci D. Şentürk M. Küfrevioğlu Ö. İ. (2011). Salicylic acid *derivatives: synthesis, features and usage as therapeutic tools*. Expert Opin. Ther. Pat. 21, 1831–1841. doi: 10.1517/13543776.2011.636354, 22098318

[ref11] Frisvad J. C. Rank C. Nielsen K. F. Larsen TO (2009). Metabolomics of Aspergillus fumigatus. Med. Mycol. 47, S53–S71. doi: 10.1080/13693780802307720 18763205

[ref12] Gonçalves C. Fernandes D. Silva I. Mateus V. (2022). Potential anti-inflammatory effect of Rosmarinus officinalis in preclinical in vivo models of inflammation. Molecules 27:609. doi: 10.3390/molecules27030609, 35163873 PMC8840442

[ref13] HDTM (2023). In vitro anti-inflammatory egg albumin denaturation assay: an enhanced approach. J. Nat. Ayurved. Med. 7, 1–6.

[ref14] Lockhart S. R. Guarner J. (2019). Emerging and Reemerging Fungal Infections.Seminars in Diagnostic Pathology. New York: Elsevier. 36, 177–181. 31010605 10.1053/j.semdp.2019.04.010 PMC11979780

[ref15] Ma Y.-M. Ma C.-C. Li T. Wang J. (2016). A new furan derivative from an endophytic aspergillus flavus of Cephalotaxus fortunei. Nat. Prod. Res. 30, 79–84. doi: 10.1080/14786419.2015.1038262, 25942282

[ref16] Miciaccia M. Belviso B. D. Iaselli M. Cingolani G. Ferorelli S. Cappellari M. . (2021). Three-dimensional structure of human cyclooxygenase (h COX)-1. Sci. Rep. 11:4312. doi: 10.1038/s41598-021-83438-z, 33619313 PMC7900114

[ref17] Peng Y. Ao M. Dong B. Jiang Y. Yu L. Chen Z. . (2021). Anti-inflammatory effects of curcumin in the inflammatory diseases: status, limitations and countermeasures. Drug Des. Devel. Ther. 15, 4503–4525. doi: 10.2147/DDDT.S327378, 34754179 PMC8572027

[ref18] Ratjen F. Bell S. C. (2021). Cystic fibrosis: anti-inflammatory therapies in the era of CFTR modulators. Lancet Respir. Med. 9, 837–849.

[ref19] Sambyal K. Singh R. V. (2021). Production of salicylic acid; a potent pharmaceutically active agent and its future prospects. Crit. Rev. Biotechnol. 41, 394–405. doi: 10.1080/07388551.2020.1869687 33618601

[ref20] Schadler D. L. George A. A. (2006). Synthesis and bioassay of a volatile fungistatic agent. Plant Health Instr. Doi.:10. doi: 10.1094/PHI-I-2006-0717-02

[ref21] Sharma D. Pramanik A. Agrawal P. K. (2016). Evaluation of bioactive secondary metabolites from endophytic fungus Pestalotiopsis neglecta BAB-5510 isolated from leaves of Cupressus torulosa D. Don. 3 Biotech 6:18. doi: 10.1007/s13205-016-0518-3 PMC5042905 28330281

[ref22] Shchegol'kov E. Shchur I. Burgart Y. V. Saloutin V. Trefilova A. Ljushina G. . (2017). Polyfluorinated salicylic acid derivatives as analogs of known drugs: synthesis, molecular docking and biological evaluation. Bioorg. Med. Chem. 25, 91–99. doi: 10.1016/j.bmc.2016.10.014, 27776888

[ref23] Su H. Kang J.-C. Cao J. Mo L. Hyde K. D. (2014). Medicinal plant endophytes produce analogous bioactive compounds. Chiang Mai J. Sci. 53, 1–12. doi: 10.12982/CMJS.2026.007

[ref24] Vecchio A. J. Malkowski M. G. (2011). The structural basis of endocannabinoid oxygenation by cyclooxygenase-2. J. Biol. Chem. 286, 20736–20745. doi: 10.1074/jbc.M111.230367, 21489986 PMC3121521

[ref25] Virshette S. Patil M. Somkuwar A. (2019). A review on medicinal plants used as anti-inflammatory agents. J Pharmacogn. Phytochem. 8, 1641–1646.

[ref26] Wu Z.-H. Li Y. Li Y. Ma M. Chen J.-L. (2018). Salicylic acid *derivatives and phenylspirodrimanes from the sponge-associated fungus Hansfordia sinuosae*. J. Asian Nat. Prod. Res. 20, 985–991. doi: 10.1080/10286020.2017.1367924, 28832193

[ref27] Yalpani N. Altier D. J. Barbour E. Cigan A. L. Scelonge C. J. (2001). Production of 6-methyl2-hydroxybenzoic acid by expression of a fungal polyketide synthase activates disease resistance in tobacco. Plant Cell 13, 1401–1410. doi: 10.1105/TPC.010015, 11402168 PMC135576

[ref28] Yatoo M. I. Gopalakrishnan A. Saxena A. Parray O. R. Tufani N. A. Chakraborty S. . (2018). Anti-inflammatory drugs and herbs with special emphasis on herbal medicines for countering inflammatory diseases and disorders—a review. Recent Patents Inflamm. Allergy Drug Discov. 12, 39–58. doi: 10.2174/1872213X12666180115153635, 29336271

